# Supplementary material for: Family-based whole-exome sequencing identifies rare variants potentially related to cutaneous melanoma predisposition in Brazilian melanoma-prone families
Source: PLoS One. 2022 Jan 27;17(1):e0262419. doi: 10.1371/journal.pone.0262419 (PMC8794197; doi:10.1371/journal.pone.0262419)
Supplement: Attachment — Submitted filename: 2021.06.18 - Response to Reviewers.pdf [file pone.0262419.s008.pdf]

A.C.Camargo Cancer Center
Centro Integrado de Diagnóstico, Tratamento, Ensino e Pesquisa

São Paulo, 18th June 2021

To Prof. Dr. Danillo G Augusto
Academic Editor, PlosOne

We are submitting a revised version of the manuscript "PONE-D-21-06922" for your reconsideration. The manuscript entitled "Family-based whole-exome sequencing identifies rare variants potentially related to cutaneous melanoma predisposition in Brazilian melanoma-prone families" was reviewed according to the suggestions made by the reviewers.

Below we present the point-to-point response to each comment and suggestion. All authors agree with the final version of the manuscript.

I hope you find it appropriate for publishing in PlosOne as a Research Article.

Very best wishes,

Dirce Maria Carraro
Genomics and Molecular Biology Group, International Research Center
A.C. Camargo Cancer Center, R. Taguá, 440, Liberdade, São Paulo – SP, Brazil.
e-mail: dirce.carraro@accamargo.org.br

Reviewer #1: A study by Fidalgo, Torrezan and colleagues analyzes rare germline variants in families with enriched cutaneous melanoma history. Unfortunately, the number of samples analyzed is small and significantly affects the power of the study, but authors preformed multiple additional analyses to understand the role of identified putative disease genes.
Several questions that need to be addressed to improve understanding of the study and ability to evaluate the results.

Response: We thank reviewer one for the careful assessment of our manuscript. We understand the concern of the reviewer regarding the small number of patients included in the study. However, we would like to clarify that by the time of patients' selection, we have attempt to contact relatives from other melanoma-prone families from our cancer registry (which included 50 individuals with clinical criteria for hereditary melanoma screened for mutations in the main associated genes), and due to the difficulty of including alive affected relatives, we were successful in including only the 10 patients from 5 families that are described in our manuscript. Also, we need to clarify that the funding we had available for performing this project was limited, which was impeditive for performing WES in more individuals, and was the reason why we have opted to focus on a family-based study of highly selected individuals. During the past months, we have tried to recruit other melanoma affected family members for 2 of the 5 families from our study, in other to improve the variant prioritization of these families; however, we were not able to recruit any additional relatives.

Although we understand the limitations that this small number of patients impose to our findings, our results represent the first WES data from melanoma-prone families in Brazil, a highly admixed population with scarce published genomic data. The identified set of rare variants with putative roles in melanoma predisposition can contribute to the future

identification of genetic similarities between patients evaluated in different studies, advancing the understanding of molecular mechanisms of melanoma carcinogenesis.

Major:
1. It is great that authors tried to validate NGS data and computational pipelines. Some additional information should be included in the manuscript to fully evaluate the validation results.

Response: We included a more detailed information regarding the validation of prioritized variants, as described in detail in question 1.2.

1.1. As stated in methods (Lines 89-92), Ion Torrent protocol was validated with GATK best practices pipeline. Could authors include a table/figure showing how concordant the results of variant calling with these pipelines were? What are the number of SNPs and Indels per sample?

Response: We have included a supplementary table (S1) containing the total number of variants called by each pipeline, as well as the concordant number of variants and unique number of variants. Briefly, the mean number of SNPs called by TVC was 33,563 and by GATK was 26,747, with a concordance mean of 26,123 SNPs. For indels, the mean number of called variants was 1,654 for TVC and 987 for GATK, with a mean concordance of 741.

1.2. Targeted NGS validation is used to validate original NGS sequencing. Conventionally, a short of variants is validated with qPCR/Sanger sequencing – to change both sequencing technology and computational pipeline. Also, coverage > 10X and VAF>20% are incomplete criteria for validation. Were the genotypes the same? It is not clear from the described approach. If so, could you please state the concordance rate?

Response: We corrected this information in the manuscript and included more detailed data regarding the validation of prioritized variants (Methods section, line 119; Result section line 168). The complete list of validate variants, their coverage, allele frequency and zygosity in both the WES and the targeted NGS sequencing is now presented in the supplementary table S3. We would like to clarify that although the criteria of coverage >10X and VAF >20% were used for setting the variant caller software, our validation data had coverage and frequencies much higher. The mean coverage of the validated variants was 16,217 (range 32 – 147,579) and the mean variant allele frequency was 0.5 (0.42 – 0.61). All variants were confirmed as heterozygous, as expected from WES data. When considering the number of candidate variants that were validated, the correct number is 66 from the 288 candidate variants (in the previous version of the manuscript we mentioned 79 variants, but 13 of these variants were excluded from our final 288 candidate variants list after being validated, due to new prioritization criteria).

2. Comparison with non-cancer exomes, used as one of the filtration steps in variant prioritization should be described in more details. Were the samples jointly called? How was systematic difference between case and control dataset assessed (e.g. using number and frequencies of common synonymous variants?

Response: The non-cancer exome samples were called individually and were provided from another study of one co-author. As in the time of our initial analysis and variant selection (performed in 2016) the ABraOM database was not available (which now contains exome sequencing data for 1,171 Brazilian healthy individuals), these 20 non-cancer individuals were used only to exclude variants that could be common in the Brazilian population and absent in

another genomic database. For this filter, we have only received a VCF file containing the variants from the non-cancer samples and compared with variants from our patients. No additional comparison was performed between these cohorts.

3. Formal statistical testing for significance of co-segregation is missing. It would be great to have any test (e.g. LOD score) performed to show how significant is co-segregation of identified variants with the phenotype, especially, given a correction for multiple test hypothesis. All the downstream pathway analyses rely on co-segregation which was not formally assessed for significance, therefore, is hard to reliably interpret.

Response: All our family duos consist of first-degree relatives, which leads to a percentage of shared variants of 50% by chance. To be able to perform a linkage analysis with significance and obtain significant LOD scores several members of the same family, with different degrees of kinship, should be evaluated. This is a recognized limitation of the study and is now better described in the discussion section of the manuscript (line 81): "*Second, all of our family duos comprised first degree relatives, which increases the number of shared variants, since any given variant has a 50% chance of being shared between the individuals, do not allow proper linkage analysis and can obscure the identification of pathogenic variants*".

Minor:
link [39] is out of order on line 73.

Response: Literature citations order was corrected in the manuscript.

Reviewer #2: The authors conducted whole exome sequencing on 10 affected individuals from 5 melanoma-prone families. The authors conducted whole exome sequencing on 10 affected individuals from 5 melanoma-prone families negative for mutations in CDKN2A and CDK4. The evaluations revealed 288 rare co-segregating coding variants across the 5 families. The authors conducted in silico, gene and pathway-based evaluations to further prioritize variants/genes for further follow-up. Based on these additional evaluations and review of the literature, the authors prioritize several potential candidate genes for further study.

Response: We thank reviewer two for the careful assessment of our manuscript. We have addressed the reviewer questions below.

Abstract. Given the small sample size in this study, the authors should modify the conclusions to indicate that the proposed genes are potential candidates. With the current study, it is not possible to conclude which genes are true candidates for melanoma genetic susceptibility.

Response: We agree with the reviewer that the identified genes are potential candidates. We have modified the abstract and parts of the discussion and conclusion to avoid overstatement of our findings.

Materials and Methods. The authors excluded variants if they were detected in all 10 samples. Although such variants would likely be sequencing artifacts or polymorphisms, there is the remote possibility that the authors might have detected a rare founder variant in their population that was responsible for disease in all 5 families. Did the authors evaluate any of the population-level rare variants observed in all 10 samples to make certain that they did not potentially reflect disease-related founder variants?

Response: We did not evaluate the presence of any of the population-level rare variants observed in all 10 samples in our first analyses. To answer the request of reviewer 2, we had re-analyzed our data and identified 5,085 variants that were present in all 10 patients. After excluding variants with MAF >0.5% in ExAc and ESP, classified as benign in Clinvar and non-coding variants (except splice site variants), there were 49 variants present in all 10 patients. 39 variants were in 2 genes that presented bad alignment in the regions of the variants. The remaining 10 variants were visually inspected, and 9 were most likely sequencing artifacts while one was most likely a variant present in one of the pseudogenes from CDC27 gene.

Depth coverage>50 was used as a filtering criteria. However, the authors report that the sequencing was conducted such that an average of 86% of the target bases were covered more than 20X. What proportion of variants were thus excluded based on depth coverage > 50 being required for retention of variants for further study?

Response: We apologized for the incorrect value in the first version of the manuscript, we corrected the coverage for 20X (methods: line 106).

Results. The authors technically validated a subset of the 288 prioritized variants. Were any family members available for further co-segregation evaluation of the prioritized variants in any of the 5 sequenced families?

Response: We have tried extensively to recruit other melanoma affected family members for 2 of the 5 families from our study that had more melanoma patients, in other to improve the variant prioritization of these families. However, unfortunately we were not able to recruit any additional affected relatives. We were able to recruit one unaffected member of one family; however, as the underlying genetic causes of melanoma predisposition in these families could be related to genes with moderate or low penetrance, it would be of limited relevance to perform the co-segregation of identified variants in unaffected family members.

Discussion. The authors acknowledge the limitations related to the small sample size of their study. Did the authors interrogate publicly available archives/databases to search for data from other melanoma cases and families to further investigate the prioritized variants/genes identified in this study?

Response: We did not analyze raw data from other melanoma studies. However, we performed an extensive literature review to identify other studies of that investigated candidate genes to melanoma predisposition. We have identified 9 studies that used comprehensive genomic approaches (WES and WGS) in melanoma patients and compared the candidate genes found in these studies (supplementary table S7) with the list of genes prioritized in our study. From our 288 initial candidates, only one the *FANCA* was identified as a candidate gene to melanoma risk in other study (Yu et al, 2018). A paragraph regarding these comparisons was added to the discission section (lines 73-78).
