# Supplementary material for: Family-based whole-exome sequencing identifies rare variants potentially related to cutaneous melanoma predisposition in Brazilian melanoma-prone families
Source: PLoS One. 2022 Jan 27;17(1):e0262419. doi: 10.1371/journal.pone.0262419 (PMC8794197; doi:10.1371/journal.pone.0262419)
Supplement: Attachment — Submitted filename: 2021.08.14 - Rebuttal letter.pdf [file pone.0262419.s009.pdf]

A.C.Camargo Cancer Center
Centro Integrado de Diagnóstico, Tratamento, Ensino e Pesquisa

São Paulo, 24th August 2021

To Prof. Dr. Danillo G Augusto
Academic Editor, PlosOne

We are submitting a revised version of the manuscript "PONE-D-21-06922" for your reconsideration. The manuscript entitled "Family-based whole-exome sequencing identifies rare variants potentially related to cutaneous melanoma predisposition in Brazilian melanoma-prone families" was reviewed according to the remaining suggestions made by the reviewers.

Below we present the point-to-point response to each comment and suggestion. All authors agree with the final version of the manuscript.

I hope you find it appropriate for publishing in PlosOne as a Research Article.

Very best wishes,

Dirce Maria Carraro
Genomics and Molecular Biology Group, International Research Center
A.C. Camargo Cancer Center, R. Taguá, 440, Liberdade, São Paulo – SP, Brazil.
e-mail: dirce.carraro@accamargo.org.br

Reviewer #1: The updated manuscript by Fidalgo, Torrezan and colleagues has been significantly improved. Clarification of the technical processing of the data aid the interpretability of the study.
For the comparison with non-cancer exomes I would still insist on performing a technical calibration of the sequencing data. Since case and control data was not jointly called technical differences between the variant calling pipelines could significantly bias the comparison. To prove the validity of this comparison, I would like to see the comparison of the synonymous variants between cases and controls. As you mentioned in your response letter, you have got the VCF file from controls with all variants. Allele frequencies comparison between cases and controls on synonymous variants could be performed with already available data relatively quickly. QQ-plot could be generated to evaluate the distribution of the test statistic.

Response: We thank reviewer one for the first and second assessment of our manuscript and the important suggestions and commentaries. We were able to perform the comparison of the allele frequencies of synonymous variants between cases and controls. As it is can be seen in these two graphics below (A: Density; B: Empirical cumulative distribution function), there is only a minor difference observed between cases and controls, which can be due to the different sequencing methods applied, different variant calling pipelines and different coverage mean, which was higher in controls than in patients. However, we believe this minor

differences do not impact the purpose of using these non-cancer exome samples, as they were used only to exclude variants that could be common in the Brazilian population and absent in another genomic database, since at that time of our first data analysis and prioritization (performed in 2016) the ABraOM database was not available (database with exome sequencing data for 1,171 Brazilian healthy individuals, that was included in a second filter of our analysis).

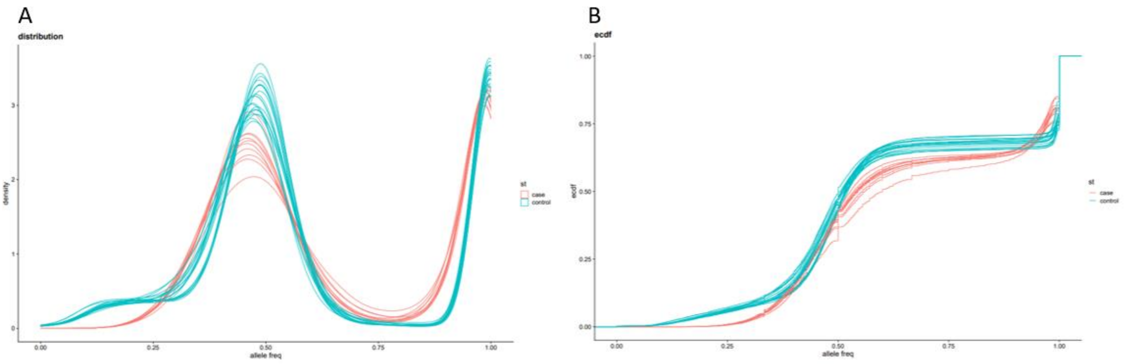

Reviewer #2: The authors attempted to address my comments. They were unable to recruit additional family members and so were not able to collect additional information on the 5 families evaluated.
There is an error in line 92 of the revision. Part of the sentence appears to be missing.
The authors should check the manuscript for grammar errors.

Response: We thank reviewer one for the first and second assessment of our manuscript and the important suggestions and commentaries.
We apologized for the missing sentence. It was corrected to "The comparison between the number of variants called by each pipeline and the numbers of concordant calls are described in the supplementary table (S1)." (methods: line 92).
We have performed a detailed revision of the manuscript to correct grammar errors.
