# Supplementary material for: Family-based whole-exome sequencing identifies rare variants potentially related to cutaneous melanoma predisposition in Brazilian melanoma-prone families
Source: PLoS One. 2022 Jan 27;17(1):e0262419. doi: 10.1371/journal.pone.0262419 (PMC8794197; doi:10.1371/journal.pone.0262419)
Supplement: Attachment — Submitted filename: 2021.12.07 - Response to reviewers.pdf [file pone.0262419.s010.pdf]

A.C.Camargo Cancer Center
Centro Integrado de Diagnóstico, Tratamento, Ensino e Pesquisa

São Paulo, 12th December 2021

To Prof. Dr. Danillo G Augusto
Academic Editor, PlosOne

We are submitting the rebuttal letter for the third review of the manuscript "PONE-D-21-06922" entitled "Family-based whole-exome sequencing identifies rare variants potentially related to cutaneous melanoma predisposition in Brazilian melanoma-prone families" for your reconsideration.

First, we would like to apologize for the delay in submitting our response. Since the reviewer requested a novel analysis for which none of the authors of this study had sufficient expertise, we had to consult other experts in the field of population genetics.

Below we present the response to the comments of the reviewers. In previous revisions, the manuscript was reviewed according to all the suggestions made the reviewers. In this version, as justified below in our letter, we have only revised the reference list to ensure that it is complete and correct, as requested by the Journal. All authors agree with the final version of the manuscript.

I hope you find it appropriate for publishing in PlosOne as a Research Article.

Very best wishes,

Dirce Maria Carraro
Genomics and Molecular Biology Group, International Research Center
A.C. Camargo Cancer Center, R. Taguá, 440, Liberdade, São Paulo – SP, Brazil.
e-mail: dirce.carraro@accamargo.org.br

Reviewer #1: The manuscript coherently outlines findings and analyses. However, I am still not convinced by the figures presented in the rebuttal letter. Since authors have allele frequencies in hand - there is a standard way of evaluating the technical bias in the data. Performing association study on synonymous variants and generating QQ-plot using obtained p-values. Genomic inflation (lambda) will become a numeric criterion for "matching" of the case and control data. I would like to kindly request this figure to be added to the supplement. Comments on the genomic inflation estimate (inflated/non-inflated) should be added to the main text.

Response: We thank reviewer one for the assessment of our manuscript. We understand the remaining concerns of the reviewer regarding the control group and the request for performing genomic inflation estimate. However, we would respectfully like to argue that,

unlike GWAS studies and other types of case-control studies that compare large populations and select candidate variants through a comparison of frequencies between cases and controls, our study has a different design. Our study is a family-based study, with the goal of identifying very rare genetic variants that are segregating in affected members of five families with high melanoma risk. In this scenario, the small number of control samples (10 Brazilian individuals) were used only to exclude variants that could be unique in the Brazilian population and absent in another genomic database, since at that time of our first data analysis and prioritization (performed in 2016) the ABraOM database was not available (database with exome sequencing data for 1,171 Brazilian healthy individuals, that was included in a second filter of our analysis). In this sense, it is of our understanding that since we did not used the controls to perform association analysis, given the small number of both controls (10 individuals) and patients (10 from 5 families), and the kinship relationship of the cases, comparing these groups with Q-Q plot analysis would not be appropriated.

Reviewer #2: All comments have been addressed.

Response: We thank reviewer one for the assessment of our manuscript and the important suggestions and commentaries in the previous rounds of revision.
